# Pseudotumoral Bladder Schistosomiasis in a 16-Year-Old Adolescent: A Case Report

**DOI:** 10.7759/cureus.105432

**Published:** 2026-03-18

**Authors:** Youness Tahri, Anouar El Moudane, Abdelghani Ouraghi, Zakariae Hayoune, Ali Barki

**Affiliations:** 1 Urology, Faculty of Medicine and Pharmacy of Oujda, Oujda, MAR; 2 Urology, Mohammed VI University Hospital, Oujda, MAR; 3 Urology, Simone Veil Hospital Center of Beauvais, Beauvais, FRA

**Keywords:** bladder mass, hematuria, schistosoma haematobium, sub-saharan africa, urinary schistosomiasis

## Abstract

We report the case of a 16-year-old adolescent from Mali who was admitted for acute macroscopic hematuria. CT urography revealed bladder wall calcifications associated with an endoluminal lesion, and cystoscopy identified a 16-mm polyp. Schistosomal serology was equivocal, while urine parasitology demonstrated nonviable eggs compatible with *Schistosoma haematobium*. A complete transurethral resection was performed, and histopathological examination revealed calcified eggs within the lamina propria without any signs of malignancy. This case highlights the importance of considering schistosomiasis in the differential diagnosis of hematuria associated with a bladder mass in children and of inquiring about prior residence in endemic areas.

## Introduction

Urogenital schistosomiasis is a chronic parasitic disease mainly caused by *Schistosoma haematobium*, which is widely distributed in sub-Saharan Africa and represents a major public health concern in endemic regions. According to the WHO, more than 200 million people are infected worldwide, with a high prevalence among children exposed to contaminated freshwater sources [1]. The pathogenesis of the disease is related to the migration of eggs through the bladder wall, inducing a granulomatous inflammatory reaction responsible for various urothelial lesions, such as wall thickening, calcifications, and polypoid formations [2]. Clinically, terminal macroscopic hematuria is the most common presentation, particularly in children and adolescents living in endemic areas. More rarely, bladder schistosomiasis may present as pseudotumoral lesions mimicking bladder tumors, which can pose a diagnostic challenge [3].

We report a case of pseudotumoral bladder schistosomiasis in a 16-year-old adolescent, presenting with macroscopic hematuria and confirmed after transurethral resection and histopathological examination.

## Case presentation

A 16-year-old adolescent with no significant medical history, originally from Mali, who had arrived in Morocco two months prior, presented to the emergency department with one day of macroscopic hematuria. On admission, the patient was conscious, afebrile, and hemodynamically stable. Clinical examination revealed suprapubic tenderness associated with nonclotting macroscopic hematuria. Post-void residual volume was zero. There was no bilateral lumbar tenderness, and examination of the external genitalia was unremarkable. The remainder of the physical examination was normal. Initial laboratory investigations showed normal renal function and an unremarkable complete blood count.

**Figure 1:**
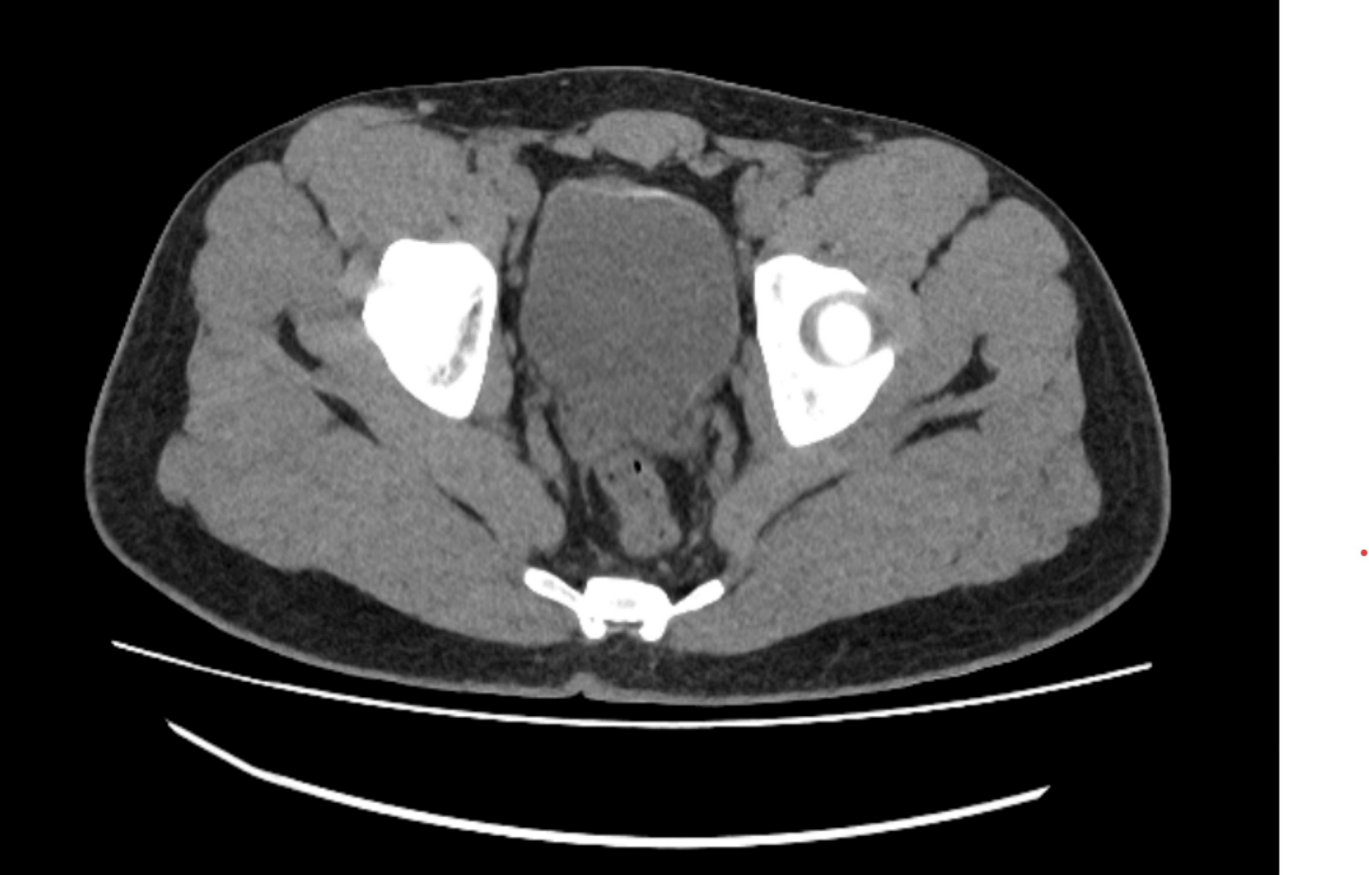
Axial non-contrast CT scan showing circumferential linear calcifications of the bladder wall

**Figure 2:**
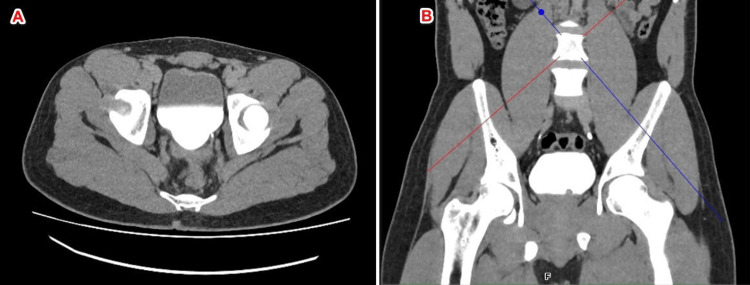
Axial (A) and coronal (B) CT images during the excretory phase (delayed images) showing a sessile filling defect measuring 16 mm in greatest diameter, arising from the left posterolateral bladder wall This lesion, suggestive of a schistosomal pseudotumor, was located on a previously calcified and altered bladder wall.

A CT urography was performed and revealed two types of abnormalities: the presence of fine calcifications of the bladder wall and an endoluminal lesion located on the left posterolateral bladder wall, measuring approximately 16 mm (Figure 1, Figure 2).

**Figure 3:**
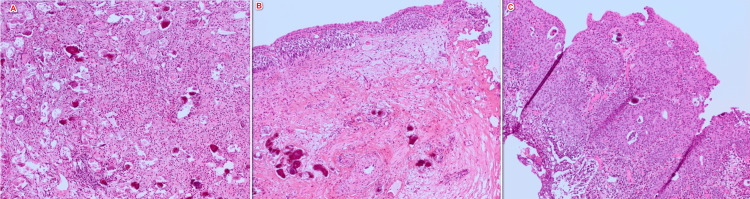
Histopathological examination of a bladder resection fragment (H&E staining, ×100) (A-C) showing multiple calcified schistosomal eggs within a fibrotic and inflammatory lamina propria

Cystoscopy revealed a single polypoid formation on the left posterolateral bladder wall, measuring approximately 16 mm in diameter. Serological testing for schistosomiasis showed equivocal results, while urine parasitological examination demonstrated the presence of nonviable eggs compatible with *S. haematobium*. He underwent complete and deep endoscopic transurethral resection of the bladder lesion. Histopathological examination revealed mildly thickened superficial urothelial fragments without architectural distortion or nuclear atypia. The underlying lamina propria was fibrotic and inflammatory, showing a polymorphic inflammatory infiltrate. Numerous calcifications corresponding to calcified schistosomal eggs were also observed, with no evidence of malignancy (Figure 3).

The patient was subsequently treated with a single oral dose of praziquantel at 40 mg/kg. The clinical outcome was favorable, with complete and sustained resolution of hematuria.

## Discussion

Urogenital schistosomiasis (urogenital bilharziasis) caused by *S. haematobium* remains endemic in sub-Saharan Africa and is a major cause of hematuria in children and adolescents. According to the WHO, it is one of the most significant neglected parasitic diseases worldwide, predominantly affecting pediatric populations living in rural areas with exposure to contaminated freshwater sources [1,2]. The pathophysiology is based on the transmural migration of eggs into the bladder lumen, inducing a chronic granulomatous reaction responsible for inflammatory, fibrotic, and calcified lesions of the bladder wall [3,4]. In our case, the patient’s Malian origin represents a key epidemiological factor. The presence of calcified eggs suggests a chronic infection acquired in an endemic area, with persistent local inflammatory activity responsible for the acute symptoms.

Clinically, terminal macroscopic hematuria is the most common manifestation in children. In endemic regions, it is considered an indirect marker of schistosomal infestation [2,3]. Advanced forms may be associated with irritative urinary symptoms, decreased bladder compliance, or upper urinary tract obstructive complications [4]. In our patient, hematuria was isolated and nonclotting, accompanied only by suprapubic tenderness, without impairment of renal function or evidence of urinary tract obstruction, suggesting a likely localized lesion.

Imaging plays a crucial role in the evaluation of schistosomal lesions. Ultrasound may reveal irregular bladder wall thickening, endoluminal masses, or hyperechoic calcifications with acoustic shadowing. CT scanning allows better characterization of bladder wall calcifications, which are often linear, diffuse, or sometimes circumferential, corresponding to chronic egg deposition. It also helps assess involvement of the upper urinary tract and detect pseudotumoral masses that may mimic neoplasia [5,6]. Pseudotumoral forms appear as enhancing intravesical soft tissue lesions after contrast administration. Radiological differentiation from primary urothelial tumors can be challenging, especially in the presence of an isolated focal lesion [5]. In our case, CT urography revealed fine diffuse bladder wall calcifications associated with a 16-mm left posterolateral endoluminal lesion. This radiological association is typically described in pseudotumoral forms of schistosomiasis and strongly supports an inflammatory parasitic etiology.

From an endoscopic standpoint, the literature describes several cystoscopic appearances, including congestive lesions, whitish granulations, inflammatory nodules, or sessile polyps. Histologically, these lesions correspond to inflammatory granulomas centered on schistosomal eggs, which may sometimes be calcified [3,6]. In our case, cystoscopy revealed a single 16-mm polyp. Although the isolated macroscopic appearance could suggest a urothelial tumor, the patient’s age and epidemiological background were more consistent with schistosomiasis. Complete transurethral resection was therefore indicated to obtain histological confirmation.

The biological diagnosis is based on the detection of eggs in urine, preferably collected at the end of micturition. Sensitivity may be reduced in old infections or in cases of low parasitic load. The presence of nonviable eggs generally reflects chronic infection. Urinary filtration techniques improve diagnostic yield, while serology may be useful when egg excretion is low, although it cannot differentiate between active and past infection [3,7]. In our patient, serology was equivocal, but parasitological examination of urine revealed nonviable eggs, confirming previous exposure. The absence of associated biological abnormalities is consistent with a localized lesion without systemic complications.

Several recent case reports in the literature have described pseudotumoral bladder lesions secondary to schistosomiasis that may mimic urothelial tumors on imaging or cystoscopy. In these cases, definitive diagnosis relied on histological analysis [8,9]. Histologically, benign lesions show preserved or mildly thickened urothelium overlying an inflammatory lamina propria containing calcified eggs. Chronic schistosomiasis is recognized as a major risk factor for squamous cell carcinoma of the bladder, related to prolonged inflammation and squamous metaplasia [10,11]. In our case, histopathological examination revealed mildly thickened urothelium without atypia, associated with a polymorphic inflammatory infiltrate and numerous calcified eggs, with no evidence of malignancy. The patient’s young age makes neoplastic transformation unlikely at this stage but warrants appropriate follow-up.

Treatment is based on praziquantel, which is recommended by the WHO and is effective against the adult forms of the parasite. In the presence of a pseudotumoral lesion, transurethral resection represents both a diagnostic and therapeutic procedure, allowing exclusion of an associated tumor [1,6]. In our patient, complete transurethral resection allowed total excision of the lesion and histological confirmation of the diagnosis, followed by appropriate antiparasitic therapy.

## Conclusions

Bladder schistosomiasis can, in rare cases, present as a pseudotumoral lesion, mimicking a bladder tumor, particularly in patients from endemic areas. The combination of hematuria, bladder wall calcifications on imaging, and polypoid lesions on cystoscopy should prompt consideration of this diagnosis, even outside endemic regions. Diagnosis relies on the detection of *S. haematobium* eggs and, most importantly, histopathological examination following lesion resection. Transurethral resection combined with appropriate antiparasitic treatment provides effective management while excluding potential malignancy. This case highlights the importance of considering epidemiological context when evaluating hematuria in young patients to avoid diagnostic errors.

## References

[1] Kokaliaris C, Garba A, Matuska M (2022). Effect of preventive chemotherapy with praziquantel on schistosomiasis among school-aged children in sub-Saharan Africa: a spatiotemporal modelling study. Lancet Infect Dis.

[2] Colley DG, Bustinduy AL, Secor WE, King CH (2014). Human schistosomiasis. Lancet.

[3] Gryseels B, Polman K, Clerinx J, Kestens L (2006). Human schistosomiasis. Lancet.

[4] Barsoum RS (2013). Urinary schistosomiasis: review. J Adv Res.

[5] Shebel HM, Elsayes KM, Abou El Atta HM, Elguindy YM, El-Diasty TA (2012). Genitourinary schistosomiasis: life cycle and radiologic-pathologic findings. Radiographics.

[6] Botelho MC, Machado JC, da Costa JM (2010). Schistosoma haematobium and bladder cancer: what lies beneath?. Virulence.

[7] Weerakoon KG, Gobert GN, Cai P, McManus DP (2015). Advances in the diagnosis of human schistosomiasis. Clin Microbiol Rev.

[8] Grech M, Busuttil G, Gauci CD, Milic M (2022). Urinary schistosomiasis: a case of late presentation. BMJ Case Rep.

[9] Darraj M (2022). Urinary bladder schistosomiasis mimicking neoplasm: a case report. Medicina (Kaunas).

[10] Mostafa MH, Sheweita SA, O'Connor PJ (1999). Relationship between schistosomiasis and bladder cancer. Clin Microbiol Rev.

[11] (1994). Schistosomes, liver flukes and Helicobacter pylori. IARC Monogr Eval Carcinog Risks Hum.

